# Ionizing Irradiation Induces Vascular Damage in the Aorta of Wild-Type Mice

**DOI:** 10.3390/cancers12103030

**Published:** 2020-10-18

**Authors:** Nobuyuki Hamada, Ki-ichiro Kawano, Farina Mohamad Yusoff, Kyoji Furukawa, Ayumu Nakashima, Makoto Maeda, Hiroshi Yasuda, Tatsuya Maruhashi, Yukihito Higashi

**Affiliations:** 1 Radiation Safety Research Center, Nuclear Technology Research Laboratory, Central Research Institute of Electric Power Industry (CRIEPI), Tokyo 201-8511, Japan; 2 Department of Cardiovascular Regeneration and Medicine, Research Institute for Radiation Biology and Medicine, Hiroshima University, Hiroshima 734-8551, Japan; kawano@hiroshima-u.ac.jp (K.-i.K.); drfarinamyusoff@hiroshima-u.ac.jp (F.M.Y.); maru0512@hiroshima-u.ac.jp (T.M.); yhigashi@hiroshima-u.ac.jp (Y.H.); 3 Biostatistics Center, Kurume University, Kurume 830-0011, Japan; furukawa_kyoji@med.kurume-u.ac.jp; 4 Department of Stem Cell Biology and Medicine, Graduate School of Biomedical and Health Sciences, Hiroshima University, Hiroshima 734-8551, Japan; ayumu@hiroshima-u.ac.jp; 5 Natural Science Center for Basic Research and Development, Hiroshima 739-8526, Japan; mmaeda@hiroshima-u.ac.jp; 6 Department of Radiation Biophysics, Research Institute for Radiation Biology and Medicine, Hiroshima University, Hiroshima 734-8551, Japan; hyasuda@hiroshima-u.ac.jp; 7 Division of Regeneration and Medicine, Medical Center for Translational and Clinical Research, Hiroshima University Hospital, Hiroshima 734-8551, Japan

**Keywords:** ionizing radiation, aorta, vascular permeability, vascular damage, inflammation

## Abstract

**Simple Summary:**

There has been renewed interest in radiation effects on the circulatory system. Here, we analyzed prelesional changes in the descending thoracic aorta of wild-type mice up to six months after a single acute exposure to 0 or 5 Gy of ^137^Cs γ-rays. We found that irradiation promoted structural disorganizations and detachment of the aortic endothelium, enhanced vascular permeability, and led to partial loss of the aortic endothelium, decreases in endothelial nitric oxide synthase, and adherens junction proteins in the aortic endothelium, and increases in inflammation and macrophage markers in the aorta. These findings suggest that irradiation causes vascular damage manifested as endothelial cell loss and increased vascular permeability, and that a decrease in adherens junction and an increase in inflammation lead to macrophage recruitment, which is thought to be involved in the early stage of atherosclerosis.

**Abstract:**

There has been a recent upsurge of interest in the effects of ionizing radiation exposure on the circulatory system, because a mounting body of epidemiological evidence suggests that irradiation induces cardio- and cerebrovascular disease at a much lower dose and lower dose rate than previously considered. The goal of our project is to determine whether dose protraction alters radiation effects on the circulatory system in a mouse model. To this end, the use of wild-type mice is pivotal albeit without manifestation of vascular diseases, because disease models (e.g., apolipoprotein E-deficient mice) are prone to hormetic responses following protracted exposures. As such, here, we first set out to analyze prelesional changes in the descending thoracic aorta of wild-type mice up to six months after a single acute exposure to 0 or 5 Gy of ^137^Cs γ-rays. Scanning electron microscopy demonstrated that irradiation facilitated structural disorganizations and detachment of the aortic endothelium. The Miles assay with an albumin-binding dye Evans Blue revealed that irradiation enhanced vascular permeability. Immunofluorescence staining showed that irradiation led to partial loss of the aortic endothelium (evidenced by a lack of adhesion molecule CD31 and 4′,6-diamidino-2-phenylindole (DAPI) signals), a decrease in endothelial nitric oxide synthase and adherens junction protein (vascular endothelial (VE)-cadherin) in the aortic endothelium, along with an increase in inflammation (tumor necrosis factor (TNF)-α) and macrophage (F4/80) markers in the aorta. These findings suggest that irradiation produces vascular damage manifested as endothelial cell loss and increased vascular permeability, and that the decreased adherens junction and the increased inflammation lead to macrophage recruitment implicated in the early stage of atherosclerosis.

## 1. Introduction

The circulatory system receives ionizing radiation at various doses and dose rates, e.g., medically (therapeutically or diagnostically) in patients and occupationally in workers. An understanding of the magnitude and mechanism of such circulatory effects of radiation is critical to gain deeper insights into normal tissue complications from radiation oncology viewpoints as well as tissue reactions (deterministic effects) from radiation protection viewpoints. Until recently, radiation effects on the circulatory system have been considered to occur only at high dose (e.g., degenerative changes and increased vascular permeability following fractionated exposures to 40–60 Gy) [1,2]. However, multiple lines of growing epidemiological evidence led the International Commission on Radiological Protection (ICRP) in 2011 recommending the first ever nominal threshold of 0.5 Gy for cardio- and cerebrovascular disease to the heart and brain, respectively, assuming that these diseases are tissue reactions with threshold-type dose–response relationships and with no dose rate effects [3]. Nevertheless, dose rate dependence, target organs or tissues, and the shape of the dose–response curve remain incompletely understood [4,5].

The goal of our project is to determine whether dose protraction changes radiation effects on the circulatory system in a mouse model. For this aim, the use of wild-type mice is critical, even though they generally experience no disease in the circulatory system. This is because disease models are prone to hormetic responses following protracted exposures particularly at a low or moderate dose, as exemplified for alleviation of atherosclerotic changes in apolipoprotein E-deficient (ApoE^−/−^) hyperlipidemic mice [6,7,8] and for delaying the onset of stroke in hypertensive rats [9] receiving chronic exposures. As a first report of this project, the present study aimed to identify potential endpoints for evaluation of prelesional changes in the descending thoracic aorta of wild-type C57BL6/J (B6J) mice following whole body exposure to 5 Gy of ^137^Cs γ-rays as a single acute dose. The aorta was selected as it represents among the proposed critical target tissues for radiation effects on the circulatory system, along with the heart and kidneys [3,10]. Selection of a dose, type of radiation, and a mouse strain was based on observations of dose protraction-dependent effects at 5 Gy of ^137^Cs γ-rays in the kidneys of B6J mice [11]. Radiotherapy patients (e.g., those with lung tumors for thoracic irradiation and re-irradiation) receive a higher total dose to the aorta, although such therapeutic doses are delivered locally in fractions [12,13]. Here, we demonstrate that ionizing irradiation induces morphological and functional damage to the thoracic aorta, which is accompanied by the elevated levels of inflammation and macrophage.

## 2. Results

**Figure 1:**
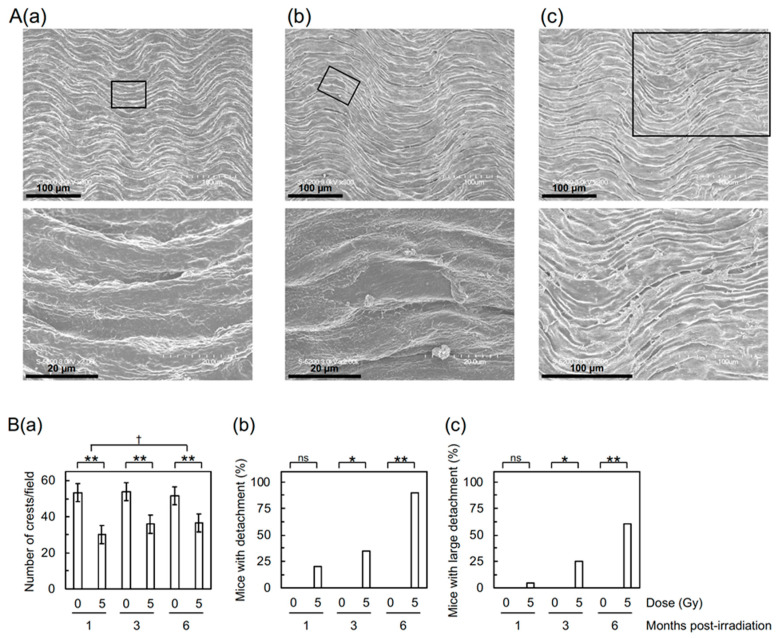
Morphological changes in the aortic endothelium of B6J mice. (**A**) Representative FE-SEM images of (**a**) normal endothelium (6 months after 0 Gy), (**b**) detachment (1 month after 5 Gy), and (**c**) large detachment (1 month after 5 Gy). Boxed areas in the upper panels are shown at higher magnification in the lower panels. Scale bars as indicated. (**B**) Quantitative analysis for (**a**) the number of crests/field (10 mice/group analyzed), and (**b**,**c**) percentage of mice with detachment and large detachment, respectively (20 mice/group analyzed). (**a**) There was a difference among three post-irradiation timepoints in irradiated groups (ANOVA *p* = 0.003, pairwise *p* < 0.02 for 1 vs. 3 months and 1 vs. 6 months) but not in sham-irradiated controls (ANOVA *p* > 0.5). ** *p* < 0.001 for irradiated vs. sham-irradiated groups at each timepoint (by the two-sample *t*-test). ^†^ The degree of difference between irradiated and sham-irradiated groups at two timepoints (pairwise *p* = 0.015). (**b**,**c**) According to the Wald test, there was a difference among three post-irradiation timepoints in irradiated groups (*p* < 0.03 for 1 vs. 6 months and 3 vs. 6 months) but not in sham-irradiated controls (*p* > 0.5). ** *p* < 0.001, * 0.001 ≤ *p* < 0.05, or ns (nonsignificant, *p* > 0.1) for irradiated vs. sham-irradiated groups at each timepoint (by Fisher’s exact test).

First, to assess the impact of irradiation on the morphology of aortic vascular endothelium, the thoracic aorta underwent field-emission scanning electron microscopy (FE-SEM) analysis. The aortic surface in sham-irradiated mice exhibited a morphology of undulations with regular repeating (Figure 1(Aa)), which was altered in irradiated mice. Irradiation reduced waviness (Figure 1(Ba)), which was considered attributable to various accompanying morphological changes, such as flattening, derangement, and cobblestone formation (Figure S1(Aa–Ac)).

**Figure 2:**
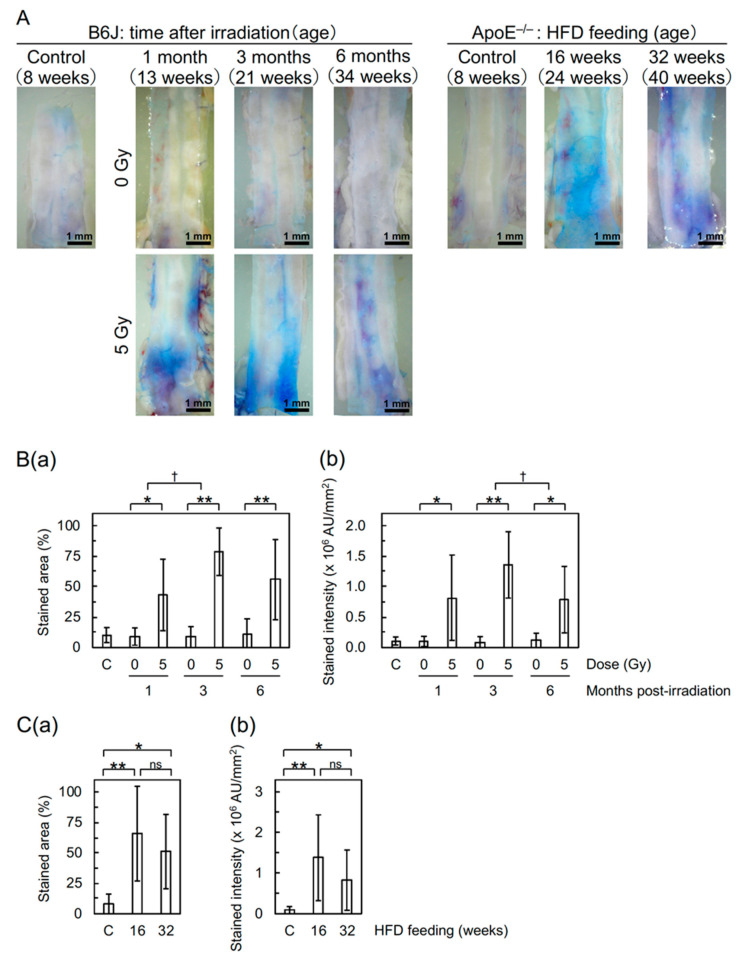
Changes in vascular permeability in the descending thoracic aorta of B6J and ApoE^−/−^ mice evaluated with the Miles assay. (**A**) Representative images of extravasated dye. (**B**) Quantitative analysis for (**a**) stained area and (**b**) intensity (**b**) in B6J mice. 10 mice/group analyzed. According to the one-way ANOVA, a difference among three post-irradiation timepoints in irradiated groups was significant for stained area (ANOVA *p* = 0.03, pairwise *p* = 0.02 for 1 vs. 3 months) and nonsignificant for stained intensity (ANOVA *p* = 0.07), but a difference among four (1 non-irradiated and 3 sham-irradiated) control groups was nonsignificant for both stained area and intensity (ANOVA *p* > 0.5). ** *p* < 0.001 or * 0.001 ≤ *p* < 0.05 for irradiated vs. sham-irradiated groups at each timepoint (by the two-sample *t*-test). ^†^ 0.001 ≤ *p* < 0.05 for the degree of a difference between irradiated and sham-irradiated groups at two timepoints (pairwise *p*). (**C**) Quantitative analysis for (**a**) stained area and (**b**) intensity (**b**) in ApoE^−/−^ mice. 10–13 mice/group analyzed. ** *p* < 0.001, * 0.001 ≤ *p* < 0.05, or ns (nonsignificant, *p* > 0.1) between two groups (by the two-sample *t*-test). A difference between non-irradiated B6J (Figure 2(Ba,Bb)) and normal-fat diet-fed ApoE^−/−^ (Figure 2(Ca,Cb)) controls was not significant (*p* > 0.5). AU, arbitrary unit. C, non-irradiated or normal-fat diet-fed controls. HFD, high-fat diet.

Detachment and large detachment of the aortic surface were observed only in irradiated mice, and its frequency increased with increasing post-irradiation time (Figure 1(Bb,Bc)). These morphometric analyses suggest that irradiation induces morphological vascular damage, which may lead to vascular dysfunction. In support of this, the Miles assay showed that irradiation increases vascular permeability in the aorta of B6J mice to an extent similar to that of high-fat diet (HFD)-fed ApoE^−/−^ mice (Figure 2), indicative of vascular dysfunction.

**Figure 3:**
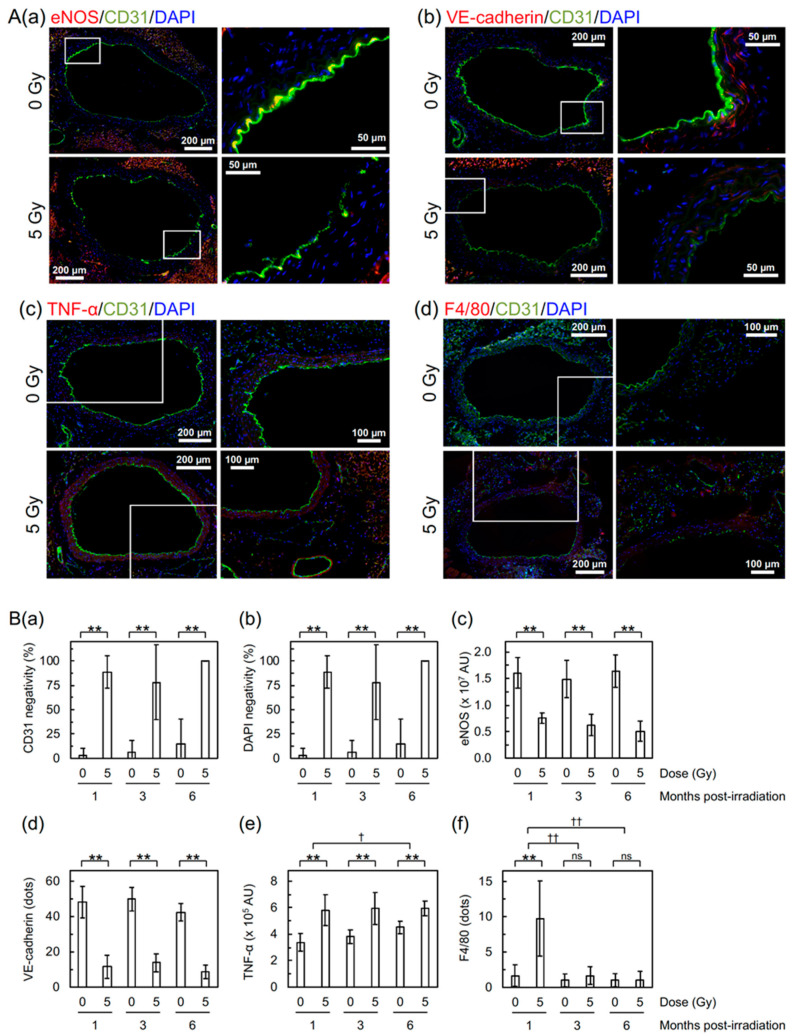
Molecular changes in the aortic endothelium of B6J mice. (**A**) Representative merged images for double immunofluorescence of CD31, and (**a**) eNOS, (**b**) VE-cadherin, (**c**) TNF-α, or (**d**) F4/80, with cell nuclei counterstained with DAPI. (**a**,**d**) 1 month post-irradiation. (**b**,**c**) 3 months post-irradiation. Upper panels, 0 Gy. Lower panels, 5 Gy. Boxed areas in the left panels (tiled images) are shown at higher magnification in the right panels. Scale bars as indicated. (**B**) Quantitative analysis for (**a**) CD31 negativity, (**b**) DAPI negativity, (**c**) eNOS, (**d**) VE-cadherin, (**e**) TNF-α, and (**f**) F4/80. For CD31 negativity, DAPI negativity and VE-cadherin, there was no difference among three post-irradiation timepoints in irradiated groups (ANOVA *p* > 0.06) and in sham-irradiated controls (ANOVA *p* > 0.1). A difference among three post-irradiation timepoints was significant for eNOS (ANOVA *p* = 0.02, pairwise *p* = 0.01 for 1 vs. 6 months) and F4/80 (ANOVA *p* = 2 × 10^−6^, pairwise *p* < 2 × 10^−5^ for 1 vs. 3 months and 1 vs. 6 months) in irradiated groups but not in sham-irradiated controls (ANOVA *p* > 0.3). For TNF-α, a difference among three post-irradiation timepoints was significant in sham-irradiated controls (ANOVA *p* = 0.0005, pairwise *p* < 0.03 for 1 vs. 6 months and 3 vs. 6 months) but not in irradiated groups (ANOVA *p* > 0.9). ** *p* < 0.001, * 0.001 ≤ *p* < 0.05, or ns (nonsignificant, *p* > 0.1) between irradiated vs. sham-irradiated groups at each timepoint (by the two-sample *t*-test). ^††^ *p* < 0.001, or ^†^ 0.001 ≤ *p* < 0.05 for the degree of a difference between irradiated and sham-irradiated groups at two timepoints (pairwise *p*). For CD31 negativity, DAPI negativity, eNOS, and VE-cadherin, there was no difference in the degree of intergroup differences between timepoints (ANOVA *p* > 0.1). 9–10 mice/group analyzed. AU, arbitrary unit.

Next, to evaluate the molecular changes, the aorta was subjected to dual immunofluorescent staining for cluster of differentiation 31 (CD31) and other markers (Figure 3A). CD31 is a marker for vascular endothelial cells (VECs), and α-smooth muscle actin (α-SMA) is a marker for vascular smooth muscle cells (VSMCs). Irradiation led to partial loss of the aortic endothelium manifested as loss of signals of CD31 and 4′,6-diamidino-2-phenylindole (DAPI) in VECs (Figure 3(Ba,Bb)), while a signal of α-SMA remains in VSMCs (Supplementary Materials Figure S3A). Irradiation also led to a decrease in endothelial nitric oxide synthase (eNOS, a marker for vascular functionality) and vascular endothelial cadherin (VE-cadherin, a marker for adherens junction) in VECs (Figure 3(Aa,Ab) and Supplementary Materials Figure S3(Bc,Bd)). These reinforce the findings on detachment and large detachment observed with the FE-SEM analysis and those on increased vascular permeability observed with the Miles assay. There was also an irradiation-induced increase in tumor necrosis factor α (TNF-α, an inflammation marker, Figure 3(Ac,Be)) in VSMCs and F4/80 (a macrophage marker, Figure 3(Ad,Bf)), suggesting that radiation-induced vascular damage induces inflammation and recruitment of macrophages.

## 3. Discussion

Here, we demonstrated that ionizing irradiation induces morphological and functional damage to the thoracic aorta, which was accompanied by the elevated levels of inflammation and macrophage.

Irradiation of B6J mice increased vascular permeability to macromolecules (e.g., albumin tested here with the Miles assay) in the aorta (to an extent similar to that in HFD-fed ApoE^−/−^ mice as shown in Figure 2) and decreased VE-cadherin (Figure 3(Bd), suggestive of disrupted adherens junction). Increased vascular permeability was also observed in the heart and kidneys of B6J mice (Supplementary Materials Figure S2), although a radiogenic increase appeared to be less evident than the aorta. These have etiological implications, because increased vascular permeability to macromolecules is one of the important steps toward development of atherosclerotic plaques, and because intercellular junctions, such as adherence and tight junctions, regulate vascular permeability. In human VECs in vitro (immortalized human coronary artery endothelial cells (HCAEC) and primary human umbilical vein endothelial cells (HUVEC)), an acute single exposure to 2–10 Gy of X-rays has recently been reported to increase endothelial permeability via degradation and internalization of VE-cadherin (mediated by ADAM10 and independent of VEGF) and also reduce tight junction protein claudin 5 [14,15]. To the best of our knowledge, this study is the first to show a radiation-induced decrease in VE-cadherin in VECs in vivo. Besides this mechanism, radiation-induced TNF-α (as shown in Figure 3(Be)) may also contribute, because TNF-α is known to increase vascular permeability via internalization and degradation of phosphorylated VE-cadherin [16]. Taken together, radiation-induced reduction of eNOS (Figure 3(Bc)) suggests endothelial dysfunction, because eNOS is pivotal for endothelial function [17].

We observed radiation-induced partial loss of the aortic endothelium with immunofluorescence. The lost area of the aortic endothelium was negative for both CD31 and DAPI (Figure 3(Ba,Bb)) but underpinning VSMCs remained (Figure S3A). This suggests loss of irradiated VECs from the aorta, to which apoptosis may contribute, at least in part, taking account of a significant increase in VECs with subcellular fragments (indicative of apoptotic bodies as a typical morphological feature of apoptosis) in the irradiated aortic endothelium (Supplementary Materials Figure S3(Ba,Bb)). The frequency of radiation-induced loss of the aortic endothelium observed with immunofluorescence and VECs with subcellular fragments were similar at 1, 3, and 6 months post-irradiation (Figure 3(Ba,Bb) and Supplementary Materials Figure S3(Bb)), whereas detachment and large detachment of the aortic epithelium observed with FE-SEM increased with increasing post-irradiation time (Figure 1(Bb,Bc)). Such a temporal difference poses a question as to whether we are looking at the same phenomena with different approaches. Our limited data make us to leave this question unanswered at this stage, but there may be at least some overlap among these phenomena.

In the tunica media of the irradiated aorta, we observed radiation-induced increases in an inflammation marker (TNF-α, Figure 3(Be)) and in VSMCs with subcellular fragments (indicative of apoptosis) (Supplementary Materials Figure S3(Bc)). Taken together, we found a radiation-induced increase in a macrophage marker (F4/80, Figure 3(Bf)) at 1 month post-irradiation, and a nonsignificant increase in leukocyte rolling on the surface of the aortic endothelium at 6 months post-irradiation (Figure S1(Ad,B)). These findings suggest that irradiation induces persistent inflammatory responses, leading to recruitment of macrophages.

The changes observed in the thoracic aorta following total body irradiation should result not only from direct effects to the thoracic aorta but also from effects from various organs/tissues (including the heart, kidneys, and other potential target organs/tissues for radiation effects on the circulatory system). As described in the introduction, the goal of our project was to determine whether dose protraction alters effects on the circulatory system. In this respect, localized thoracic or heart irradiation is feasible for acute and fractionated irradiation but is impractical for chronic irradiation. Therefore, to make inter-comparisons of effects among different irradiation conditions (e.g., acute, fractionated, and chronic) easier, we decided to consistently use total body irradiation.

Undoubtedly, the findings obtained with ApoE^−/−^ mice have contributed greatly to a better understanding of the mechanisms underlying radiation effects on the circulatory system, as recently reviewed [18]. However, the shape of the dose–response relationship and the impact of low-dose-rate irradiation in ApoE^−/−^ mice remain uncertain. For instance, Mitchel et al. observed protective effects of low-dose-rate irradiation at 0.025–0.5 Gy [6], whereas Mancuso et al. observed sparing dose rate effects (with more predominant effects in the descending thoracic aorta following low-dose-rate irradiation than those following high-dose-rate irradiation) at 0.3 Gy but little dose rate effects at 6 Gy [19], both suggesting a non-linear dose response [6,19]. Considering such uncertainty, we used ApoE^−/−^ mice only as positive controls for effects on the circulatory system, with no irradiation experiments (e.g., irradiation of mice fed a normal- or high-fat diet) performed. Nevertheless, the present data showed that irradiation increases vascular permeability in the aorta of B6J mice to an extent similar to that in HFD-fed ApoE^−/−^ mice (Figure 2). To examine the underlying mechanisms, we plan to perform analysis of the ApoE^−/−^ aorta using FE-SEM and immunofluorescence.

In conclusion, we demonstrated that acute single exposure to ionizing radiation induces vascular damage and dysfunction. It remains unclear whether the phenomena and mechanisms for radiation effects on the circulatory system differ with dose protraction [3,10]. With the endpoints identified in the present study for evaluation of prelesional changes, further experiments are underway to compare the effects of acute, fractionated, and chronic exposures in irradiated B6J mice.

## 4. Materials and Methods

### 4.1. Mice

Male B6J mice purchased at 7 weeks of age from Charles River Laboratories Japan were acclimated for a week before starting experiments. ApoE^−/−^ mice on the B6J genetic background were purchased from Charles River Laboratories Japan, bred in-house, and males were used for experiments. Mice were maintained under a 12-h light/dark cycle (light onset at 8 am) with ad libitum access to food and water. All mice were fed a normal-fat diet (NFD, ~3.5 kcal/g, ~13% of the calorie from crude fat, MF obtained from Oriental Yeast, Japan), except that ApoE^−/−^ mice aged 8 weeks were fed a high-fat diet (HFD, ~5 kcal/g, ~60% of the calorie from crude fat, HFD-60 from Oriental Yeast) for 16 or 32 weeks. All animal experiments were approved by the Institutional Animal Care and Use Committee of Hiroshima University (approval number A16–139), and carried out in compliance with the guidelines of the Institute of Laboratory Animal Science, Hiroshima University.

### 4.2. Irradiation

Just prior to irradiation, 10 unanesthetized B6J mice at age 8 weeks were placed in a 12-compartment pie cage (Natsume Seisakusho, Japan), and exposed to a single acute dose of ^137^Cs γ-rays at 5 Gy from Gammacell 40 Exactor at a dose rate of 0.5 Gy/min. Sham-irradiated controls were handled in parallel with the test mice. At 1, 3, or 6 months after irradiation (viz, at age 13, 21, and 34 weeks, respectively), some mice were subjected to a Miles assay with an albumin-binding dye Evans blue to analyze vascular permeability, whereas tissue samples were taken from other mice anesthetized with isoflurane and perfused transcardially with phosphate-buffered saline (PBS^−^). Of the collected descending thoracic aorta, the cranial half was evaluated for morphological changes with field-emission scanning electron microscopy (FE-SEM), the caudal half being assessed for cellular and molecular changes with double immunofluorescence staining. No mice died or appeared moribund throughout the observation period, although irradiated mice exhibited a slight but statistically significant decrease in body weight compared with sham-irradiated mice.

### 4.3. Miles Assay

Anesthetized mice were intravenously injected with 100 µL of 1% Evans blue dye, and perfused transcardially with PBS^−^ 40 min later, followed by tissue sampling. The descending thoracic aorta was opened longitudinally and washed with PBS^−^. Images captured with the Dino-Lite AM4113 microscope were used to quantify staining (in terms of stained area and stained intensity) in the aorta with ImageJ (version 1.52a). The heart (after being opened, weighed, and washed with PBS^−^) and kidneys (after being weighed) were incubated at 56 °C in formamide to elute the dye, and the optical density at 610 nm (OD_610_) in the supernatant was measured. Using a standard curve, the OD_610_ values were changed to dye (µg) captured per tissue (g).

### 4.4. FE-SEM

The cranial half of the descending thoracic aorta was opened longitudinally, washed with PBS^−^, fixed in 1% glutaraldehyde, washed with PBS^−^, and dehydrated in an ethanol series (30%, 50%, 70%, 90%, 99.5%, and 100%). The FE-SEM (Hitachi High-Technologies S-5200) was operated at 3 kV, and the entire area of the carbon-coated tissue was observed. The surface of the normal aortic endothelium exhibited less frequent horizontal waves and more frequent vertical waves (Figure 1(Aa)), and the number of crests in such vertical waves was counted in each of the seven fields/mouse (each field corresponds to the entire area of the image taken at 300 × magnification, 1 crest/field corresponding to ~7.5 crests/mm^2^). The detached area in the aortic endothelium with sizes in the longer axis of a few tens of microns was designated “detachment” (Figure 1(Ab)) and that of the order of 100 µm was designated “large detachment” (Figure 1(Ac)). Each mouse was considered positive if one or more such areas existed in the aorta, and such positivity in the group was evaluated independent of the number of such areas in each mouse (this was also the case for leukocyte rolling).

### 4.5. Immunofluorescence

The caudal half of the descending thoracic aorta was embedded in optimal cutting temperature (OCT) compound (Sakura Finetek, Japan), snap frozen (first with the cold isopentane and then liquid nitrogen), and stored at −80 °C. The tissue was transversally cryosectioned at a 5-µm thickness (Leica CM1950). The section was mounted onto a glass slide coated with 3-aminopropyltriethoxysilane (Matsunami, Japan), air-dried, fixed in 4% paraformaldehyde, washed with PBS^−^, blocked with Block Ace (Yukijirushi, Japan), reacted with primary antibodies (diluted 1:100 or 1:200), washed with PBS^−^, and reacted with secondary antibodies (diluted 1:500), with cell nuclei counterstained with 4′,6-diamidino-2-phenylindole (DAPI). Primary antibodies used were rabbit polyclonal against VE-cadherin (also called cadherin-5 or CD144) and TNF-α, rabbit monoclonal against eNOS (Clone D9A5L) and F4/80 (Clone SP115), rat monoclonal against CD31 (also called PECAM-1, Clone MEC 13.3), and mouse monoclonal againstα-SMA (clone 1A4, Cy3 conjugated). Secondary antibodies used were anti-rabbit IgG conjugated with Alexa Fluor 594, and anti-rat IgG conjugated with Alexa Fluor 488. The sections were simultaneously reacted with two different primary antibodies (a set of CD31 stained red and one of the five antibodies stained green), and then with two different secondary antibodies (anti-rabbit and anti-rat, except for α-SMA). Primary antibodies were purchased from Abcam (except for α-SMA from Sigma, eNOS from Cell Signaling Technology, and CD31 from BD Pharmingen), and secondary antibodies from Invitrogen. Images were captured using a Keyence BZ-9000 fluorescence microscope (Keyence, Japan), and processed with Keyence BZ-X Analyzer. For each mouse, staining was quantified as follows: intensity of red signals in randomly selected five areas (150 µm × 50 µm) in the tunica intima in one image taken at 60 × magnification for eNOS, the number of dots in the tunica intima in four images taken at 60 × magnification for VE-cadherin, intensity of red signals in randomly selected five areas (50 µm × 50 µm) in the tunica media in one image taken at 60 × magnification for TNF-α, the number of dots in the entire aortic area (including the tunica adventitia) in four images taken at 20 × magnification for F4/80, and loss of signals in 6 tiled images for CD31 and DAPI. For cells with subcellular fragments, all aortic VECs in the tunica intima and aortic VSMCs in the tunica media were counted at 60 × magnification in two sections for each mouse (total of 92–285 VECs and 332–1242 VSMCs counted/mouse in two sections each stained for eNOS/CD31 and VE-cadherin/CD31 with nuclei counterstained with DAPI). A tiled image for the cross section of the aortic wall was created from 30–60 images for each of three colors (red, green, and blue) taken at 60 × magnification for eNOS and VE-cadherin, and from 8–12 images taken at 20 × magnification for other markers.

### 4.6. Statistical Analysis

Statistical analyses were performed using R statistical software (version 3.6.1, R Foundation, https://www.r-project.org/), where *p* < 0.05 was considered significant (*p* < 0.001 presented as double symbols ** or ^††^, 0.001 ≤ *p* < 0.05 as single symbols * or ^†^). Asterisks were used for intra-timepoint comparisons (e.g., irradiated vs. sham-irradiated groups at each timepoint), whereas daggers were used for inter-timepoint comparisons of such intra-timepoint differences (the degree of differences in irradiated and sham-irradiated groups between two timepoints). *p* values determined by the one-way analysis of variance (ANOVA) using the F-test of homogeneity among the group means are presented as ANOVA *p*. *p* values determined by pairwise comparisons using the *t*-tests with Bonferroni corrections are presented as pairwise *p*. *p* values determined by the two-sample (Student’s) *t*-test for the null hypothesis of equal means, Fisher’s exact test, and the Wald test (logistic regression) are presented as *p*. The statistical test used is described in each figure legend. Each data point was obtained from 9–13 mice and represents means and standard deviations, unless otherwise specified.

## 5. Conclusions

The present results suggest that a single acute exposure to 5 Gy of ^137^Cs γ-rays at a dose rate of 0.5 Gy/min produces vascular damage manifested as endothelial cell loss and increased vascular permeability, and that the decreased adherens junction and the increased inflammation lead to macrophage recruitment, which is thought to be involved in the early stage of atherosclerosis.

## Supplementary Materials

The following are available online at https://www.mdpi.com/2072-6694/12/10/3030/s1, Figure S1: Morphological changes in the aortic endothelium of B6J mice, Figure S2: Changes in vascular permeability in the heart (A) and kidneys (B) of B6J mice examined with the Miles assay, Figure S3: Molecular and nuclear changes in the aortic endothelium of B6J mice.
