# Supplementary material for: Ionizing Irradiation Induces Vascular Damage in the Aorta of Wild-Type Mice
Source: Cancers (Basel). 2020 Oct 18;12(10):3030. doi: 10.3390/cancers12103030 (PMC7603246; doi:10.3390/cancers12103030)
Supplement: Supplementary file 1 [file cancers-12-03030-s001.pdf]

Supplementary Materials

Ionizing Irradiation Induces Vascular Damage in the Aorta of Wild-Type Mice

Nobuyuki Hamada, Ki-ichiro Kawano, Farina Mohamad Yusoff, Kyoji Furukawa, Ayumu Nakashima, Makoto Maeda, Hiroshi Yasuda, Tatsuya Maruhashi and Yukihito Higashi

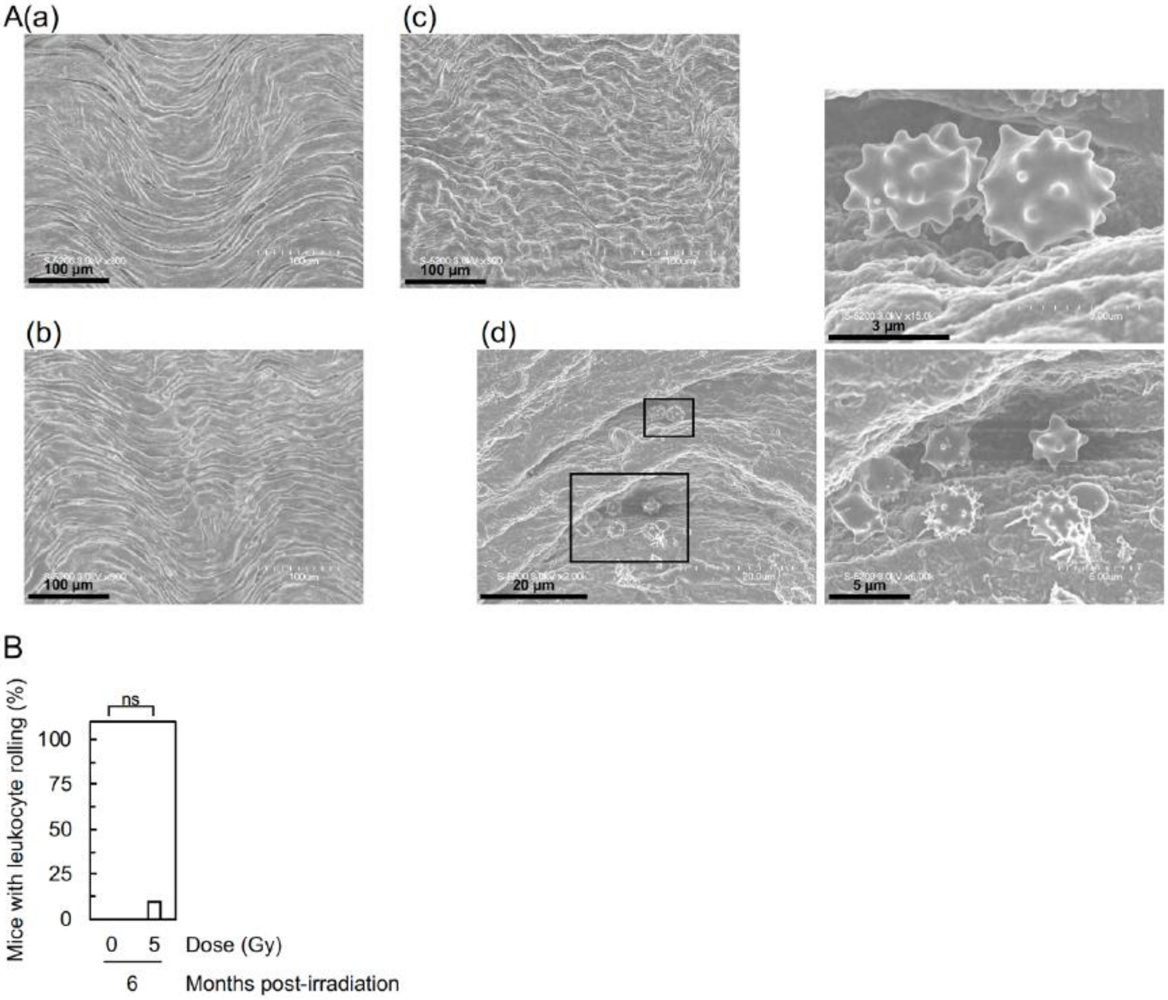

Figure S1. Morphological changes in the aortic endothelium of B6J mice. **A.** Representative FE-SEM images of (**a**) flattened endothelium (6 months after 5 Gy), (**b**) deranged endothelium (6 months after 5 Gy), (**c**) cobblestone-shaped endothelium (1 month after 5 Gy), and (**d**) endothelium with rolling leukocytes (6 months after 5 Gy). Scale bars as indicated. (**d**) Upper and lower boxed areas in the left panel are expanded to the corresponding right panels. **B.** Quantitative analysis for percentage of mice with leukocyte rolling (10 mice/group analyzed). ns, nonsignificant at $p > 0.5$ (by Fisher's exact test).

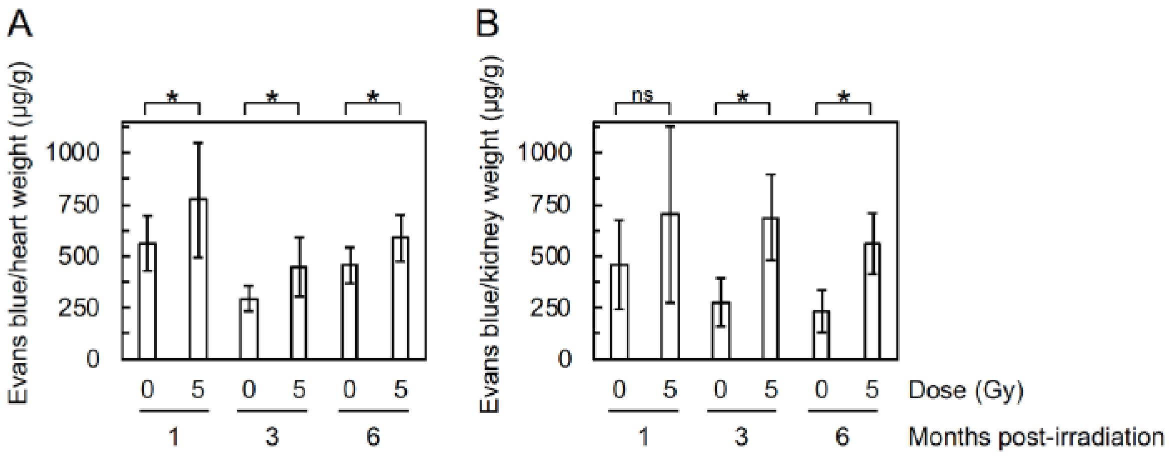

Figure S2. Changes in vascular permeability in the heart (**A**) and kidneys (**B**) of B6J mice examined with the Miles assay (9–10 mice/group analyzed). In the heart, there was a difference among three post-irradiation timepoints in sham-irradiated controls (ANOVA $p = 8 \times 10^{-6}$, pairwise $p < 0.004$ for 1 vs. 3 months and 3 vs. 6 months) and in irradiated groups (ANOVA $p = 0.005$, pairwise $p = 0.004$ for 1 vs. 3 months). In kidneys, there was a difference among three post-irradiation timepoints in sham-irradiated controls (ANOVA $p = 0.01$, pairwise $p < 0.05$ for 1 vs. 3 months and 1 vs. 6 months) but not in irradiated groups (ANOVA $p > 0.5$). **$0.001 \leq p < 0.05$, or ns (nonsignificant, $p = 0.13$) for irradiated vs. sham-irradiated groups at each timepoint (by the two-sample t-test). Both in the heart and kidneys, there was no difference in the degree of intergroup differences between timepoints (ANOVA $p > 0.5$).

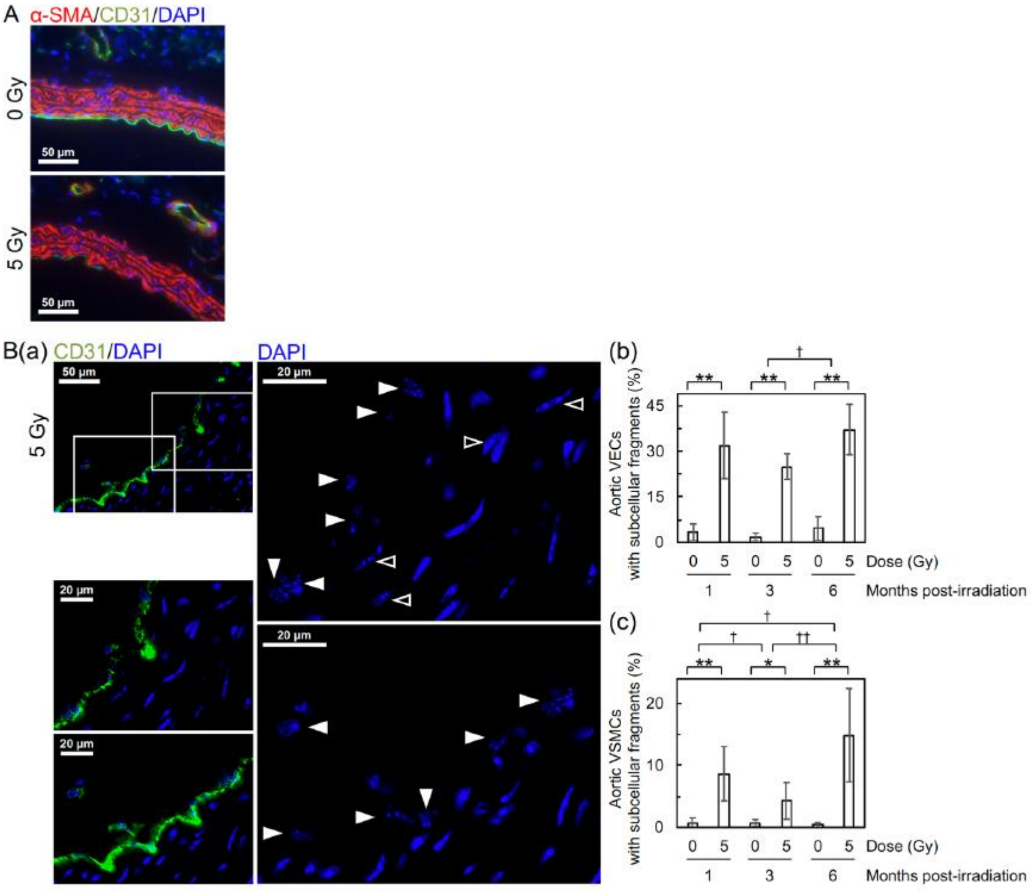

Figure S3. Molecular and nuclear changes in the aortic endothelium of B6J mice. **A**. Representative merged immunofluorescence images for α-SMA, CD31, and DAPI. Scale bars as indicated. **B**. Cells with subcellular fragments in the aorta. (**a**) Representative immunofluorescence images. The left

upper panel is the same as the lower right panel in Fig. 3Aa (i.e., at 1 month after 5 Gy), but without eNOS. Images in the left panels are merged for CD31 and DAPI. Upper and lower boxed areas in the upper left panel are shown at higher magnification in the middle and lower left panels, respectively. Images in the middle and lower left panels, respectively, are further enlarged in the upper and lower panels but without CD31. Scale bars as indicated. Closed and open arrowheads point to vascular endothelial cells (VECs) with subcellular fragments in the tunica intima and vascular smooth muscle cells (VSMCs) with subcellular fragments in the tunica media, respectively. Quantitative analysis for (**b**) aortic VECs with subcellular fragments and (**c**) aortic VSMCs with subcellular fragments (9–10 mice/group analyzed). A difference among three post-irradiation timepoints was significant in irradiated groups (ANOVA $p < 0.01$, pairwise $p = 0.008$ and 0.0004 for 3 vs. 6 months in VECs and VSMCs, respectively) but not in sham-irradiated controls (ANOVA $p > 0.1$). $^{**}p < 0.001$, or $^{*}0.001 \leq p < 0.05$ for irradiated vs. sham-irradiated groups at each timepoint (by the two-sample t-test). $^{††}p < 0.001$, or $^{†}0.001 \leq p < 0.05$ for the degree of a difference between irradiated and sham-irradiated groups at two timepoints (pairwise p).

© 2020 by the authors. Licensee MDPI, Basel, Switzerland. This article is an open access article distributed under the terms and conditions of the Creative Commons Attribution (CC BY) license (http://creativecommons.org/licenses/by/4.0/).
